# Enhancement of CO_2_ capture performance of aqueous MEA by mixing with [NH_2_e-mim][BF_4_]

**DOI:** 10.1039/c7ra11757d

**Published:** 2018-01-10

**Authors:** Mei Wang, Mingming Wang, Na Rao, Jiale Li, Jianfen Li

**Affiliations:** School of Chemical and Environmental Engineering, Wuhan Polytechnic University Wuhan Hubei 430023 PR China wangmei0223@hotmail.com lijfen@163.com +86 27 83963954

## Abstract

Alcohol amine solutions have a high absorption capacity and rate for CO_2_ capture, however, there are some shortcomings such as high energy-consumption and low stability. To enhance CO_2_ capture performance of aqueous MEA, a functional ionic liquid ([NH_2_e-mim][BF_4_]) was introduced based on the advantages for CO_2_ capture. Absorbents were prepared with the molar concentration ratio of [NH_2_e-mim][BF_4_] to the 30 vol% aqueous MEA of 0 : 10, 1 : 9, 2 : 8, 3 : 7, 4 : 6 and 6 : 4. The density and the viscosity of the investigated absorbents were measured and the effects of the molar fraction of [NH_2_e-mim][BF_4_] (*n*_I_) and temperature on CO_2_ absorption performance were investigated. CO_2_ desorption performance of the solvent at different temperatures was discussed. The stability performance of the absorbent with *n*_I_ of 2 : 8 (I/M_2:8_) was examined by five consecutive cyclic tests. The results showed that for pure CO_2_, the I/M_2:8_ displayed the highest absorption performance at 303 K under 1 bar: a comparable CO_2_ absorption capacity of the 30 vol% aqueous MEA and a higher CO_2_ absorption rate at the later absorption stage. Moreover, with the increase of temperature, CO_2_ absorption capacity and rate decreased, while CO_2_ desorption efficiency and rate increased. 393 K was chosen as the optimum desorption temperature with the desorption efficiency of 99.31%. The introducing of IL contributed to CO_2_ desorption performance of the absorbents significantly. The properties (CO_2_ absorption capacity, mass loss, density and viscosity) of the I/M_2:8_ during the cycles suggested that the IL-MEA mixture had an excellent stability performance.

## Abstract 2

Alcohol amine solutions have a high absorption capacity and rate for CO_2_ capture, however, there are some shortcomings such as high energy-consumption and low stability.

## Graphical abstract

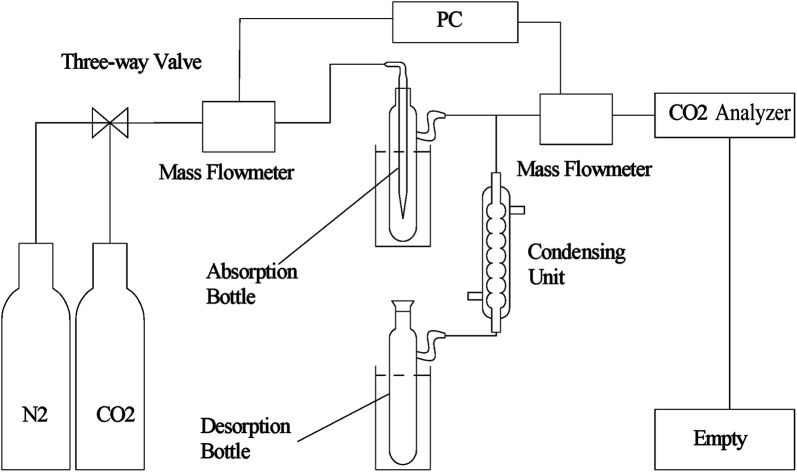

## 1. Introduction

Global warming, caused by excessive emission of carbon dioxide (CO_2_), has become one of the world's major environmental issues.^1–4^ The reduction of CO_2_ emissions by the capture of CO_2_ from flue gases is considered as an effective method to mitigate the greenhouse effect.^5–7^ Currently, the leading technology involves chemical absorption with aqueous amine solutions (typically 30 vol% amine by volume).^8,9^ However, the commercially available aqueous amine solutions, represented by monoethanolamine (MEA), present many disadvantages including high regenerative energy and degradation in the presence of oxygen.^10–12^ Moreover, the volatilization of amines causes environmental pollution and corrosion, as well as raises the cost of operation and amortized installation.^13,14^

In recent years, considerable research efforts have been made to study the capture performance of the solvents that could overcome the aforementioned disadvantages. Ionic liquids (ILs), which are salts with a melting point below 100 °C and very low volatility, have great promise in the near future considering their high CO_2_ capture performance and reutilization.^15–19^ Among these, functionalized ILs, which could simultaneously improve absorption rate and selectivity of CO_2_ capture through the reversible reactions between reactive group of the ILs and CO_2_, have been intensively investigated in the past several decades.^1,20–22^ Bates *et al.* synthesized the IL (1-(1-aminopropyl)-3-butylimidazole fluoroborate, [NH_2_p-bim][BF_4_]) with amine moieties as the functional groups.^23^ The IL shows a high adsorption capacity of 0.5 mol CO_2_ per mol IL. In addition, some other studies also found that the amine-functionalized ILs have high CO_2_ capture performance.^24–27^ However, the high viscosity of this amine-functionalized ILs influenced the mass transfer between the liquids and the gas seriously, resulting in a low CO_2_ absorption capacity.^28–32^

In this paper, a new kind of solvent was developed by mixing an amine-functionalized IL (1-(1-aminoethyl)-3-methylimidazole fluoroborate [NH_2_e-mim][BF_4_]), with 30 vol% aqueous MEA solutions to investigate whether there was a synergetic effect on CO_2_ capture performance. The effects of the molar concentration ratio of [NH_2_e-mim][BF_4_] to MEA in the mixture and the temperature on CO_2_ absorption performance of the solvents were explored. Further, the desorption performance of the solvents at different temperature were discussed. Moreover, the cyclic stability of the absorbents were evaluated by five consecutive CO_2_ absorption–desorption tests.

## 2. Experimental

### Materials

CO_2_ (purity ≥ 99.99%) was purchased from Wuhan Minghui gas Co., Ltd., China. Ethanolamine (MEA, AR) and sodium borate (NaBF_4_, CP) were purchased from Sinopharm Chemical Reagent Co., Ltd., China. 2-Bromine ethylamine hydrobromide (C_2_H_7_Br_2_N, 98%) and 1-methylimidazole (C_4_H_6_N_2_, 99%) were provided by Aladdin Reagent Co., Ltd., China.

### Characterization and measurement

#### Preparation of the investigated solvents

[NH_2_e-mim][BF_4_] was synthesized and the purity was measured according to our previous work.^22,33,34^

An appropriate amount of material was taken in a closed flask according to the molar concentration ratio of the [NH_2_e-mim][BF_4_] to the 30 vol% aqueous MEA of 0 : 10, 1 : 9, 2 : 8, 3 : 7, 4 : 6 and 6 : 4. The corresponding molar fraction of [NH_2_e-mim][BF_4_] (*n*_I_) was 0, 0.1, 0.2, 0.3, 0.4, 0.6. It was mixed evenly by ultrasonic vibration.

#### Measurement of physical properties

The density was measured using a density meter (DM45 Delta Range, Mettler Toledo of Switzerland) that operated *via* electromagnetically induced oscillation of a glass U-form tube, with automatic compensation for variations in atmospheric pressure. The accuracy of the density meter measurements was ±0.00005 g cm^−3^ for all operating conditions.

The viscosity was measured using a viscometer (DV-II+ Pro, Brookfield of USA). The “ULA” spindle and jacketed sample cell were used for these relatively low viscosity absorbents. The accuracy of viscometer was ±1% of the reading for torque measurement with a repeatability of ±0.2% of the reading. The temperature of the jacketed sample chamber was controlled *via* a circulating bath (TC-602P, Brookfield of USA) with a temperature stability of ±0.01 K.

The mass of the solution was measured by a precision electronic balance (AR2140, Mettler Toledo of Switzerland). The accuracy of the mass measurements was ±0.00001 g.

#### Determination of CO_2_ absorption/desorption performance

**Fig. 1:**
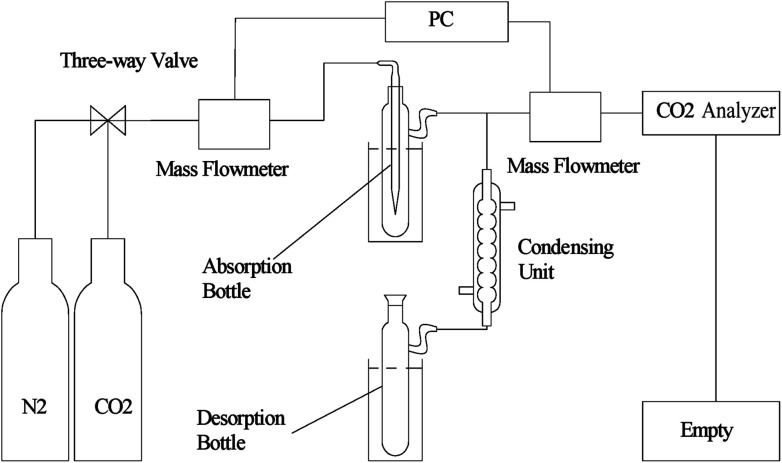
Experimental apparatus for CO_2_ absorption/desorption.

CO_2_ absorption and desorption performance of the solvents were measured by a homemade apparatus as shown as Fig. 1. The error between the measured CO_2_ solubility and the theoretical value is less than 5%.

For CO_2_ absorption experiment, CO_2_ intake speed was controlled at approximately 60 mL min^−1^ under 1 bar. CO_2_ intake and outlet flow was determined by mass flow meters (50L type, SIERRA Flow Measurement and Control Technology Company of USA) with measurement error range of ±1%, which was recorded by a computer every 30 s. CO_2_ intake flow was recorded as *V*_*i*_ and *V*′_*i*_ for CO_2_ outlet flow at time “*i*”. The volume of CO_2_ solubility in the investigated solvents (Δ*V*_*i*_, mL) could be obtained by eqn (1). CO_2_ absorption volume at time “*t*” (*Q*_a_, mL) and the molar fraction of CO_2_ (*X*_CO_2__, unit: mol CO_2_ per mol mixture, expressed as mol per mol) in the solvents was calculated from eqn (2) and (3).

1 Δ*V*_*i*_ = *V*_*i*_ − *V*′_*i*_

2
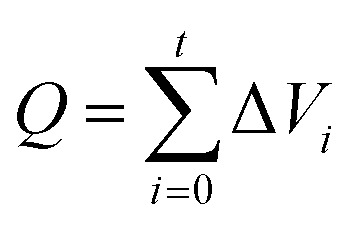
3
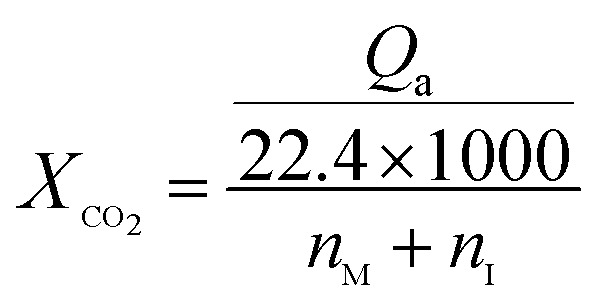
where *n*_M_ and *n*_I_ was the molar amount of MEA and [NH_2_e-mim][BF_4_] in the investigated absorbents.

During CO_2_ desorption, temperature was regulated to range from 383 K to 398 K. CO_2_ liberation volume was measured by a mass flowmeter and recorded in a computer. CO_2_ desorption capacity (*Q*_d_, mL) was also calculated by eqn (2). And CO_2_ desorption efficiency (*η*, %) was defined as the percentage of CO_2_ desorption capacity to corresponding CO_2_ absorption capacity, which could be calculated by eqn (4).4
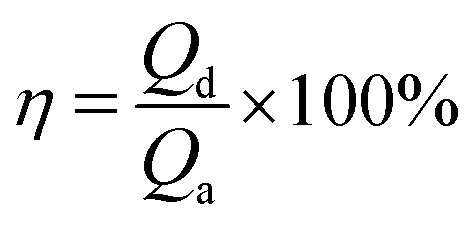


The investigated solvents were subjected to the steps previously mentioned to carry out CO_2_ absorption–desorption cycle experiments.

## 3. Results and discussion

### Physical properties of the investigated solvents

**Fig. 2:**
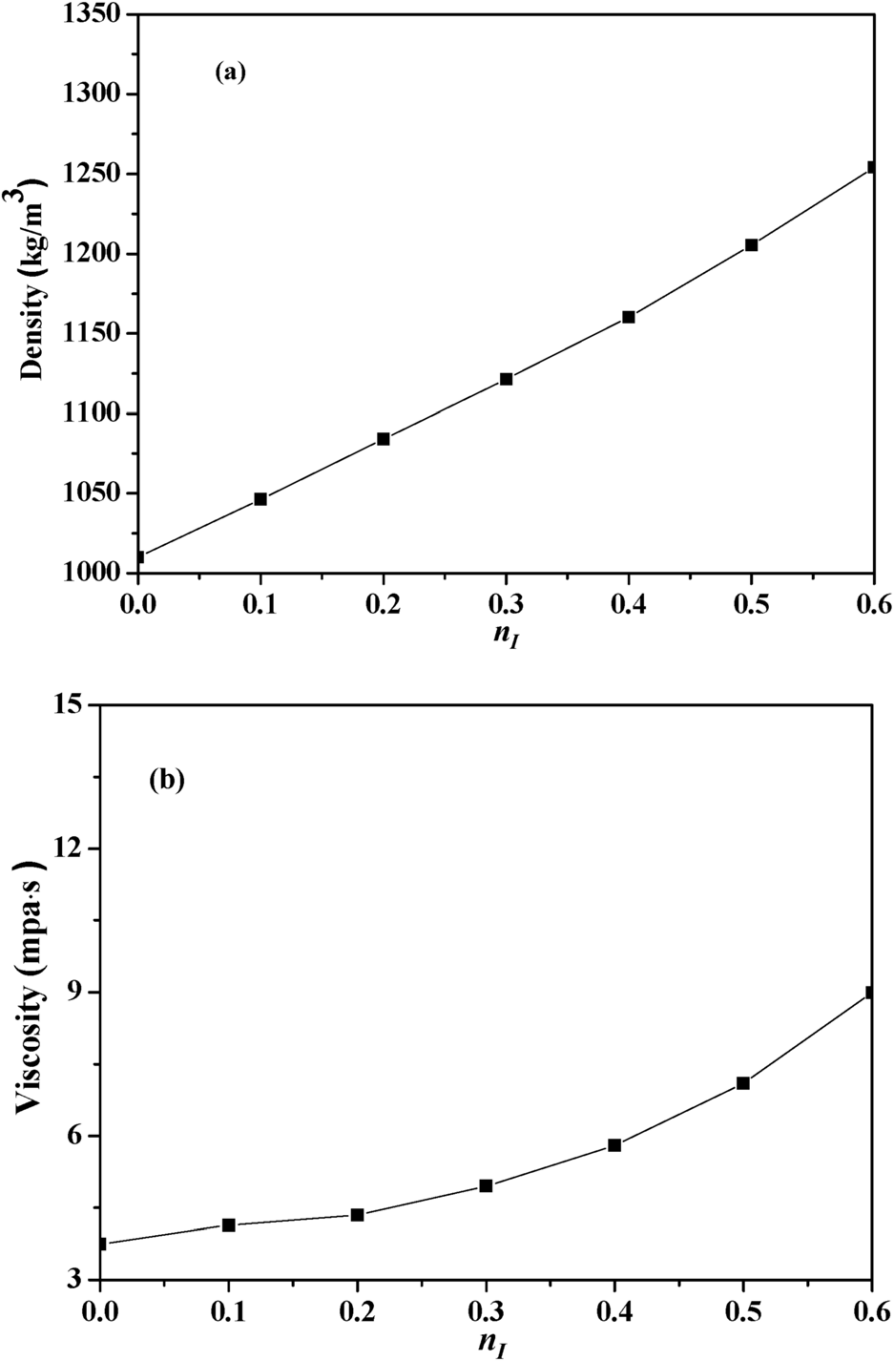
Physical properties of the absorbents with different *n*_I_ at 303 K: (a) density; (b) viscosity.

Density and viscosity of the investigated solvents at 303 K under 1 bar were shown in Fig. 2.

The density value of the mixed absorbents was between the density value of 30 vol% solution (1007.2 kg m^−3^) and the [NH_2_e-mim][BF_4_] (1472.2 kg m^−3^) which were measured at the same condition. And the viscosity of the mixed absorbents also showed the same law. The viscosity of [NH_2_e-mim][BF_4_] was 3589.2 mPa s determined by our group,^22,34^ while the viscosity of the absorbents was below 10 mPa s with *n*_I_ of 0–0.6, which showed that the addition of 30 vol% solution reduced the viscosity of absorbents significantly. According to Fig. 2, both the density and the viscosity of the investigated absorbents increased with the increase of *n*_I_. The density was basically linear related to *n*_I_ and the slope of viscosity kept increasing with the increase of *n*_I_ when *n*_I_ exceed 0.2.

### Effects of the *n*_I_ on the absorption performance

**Fig. 3:**
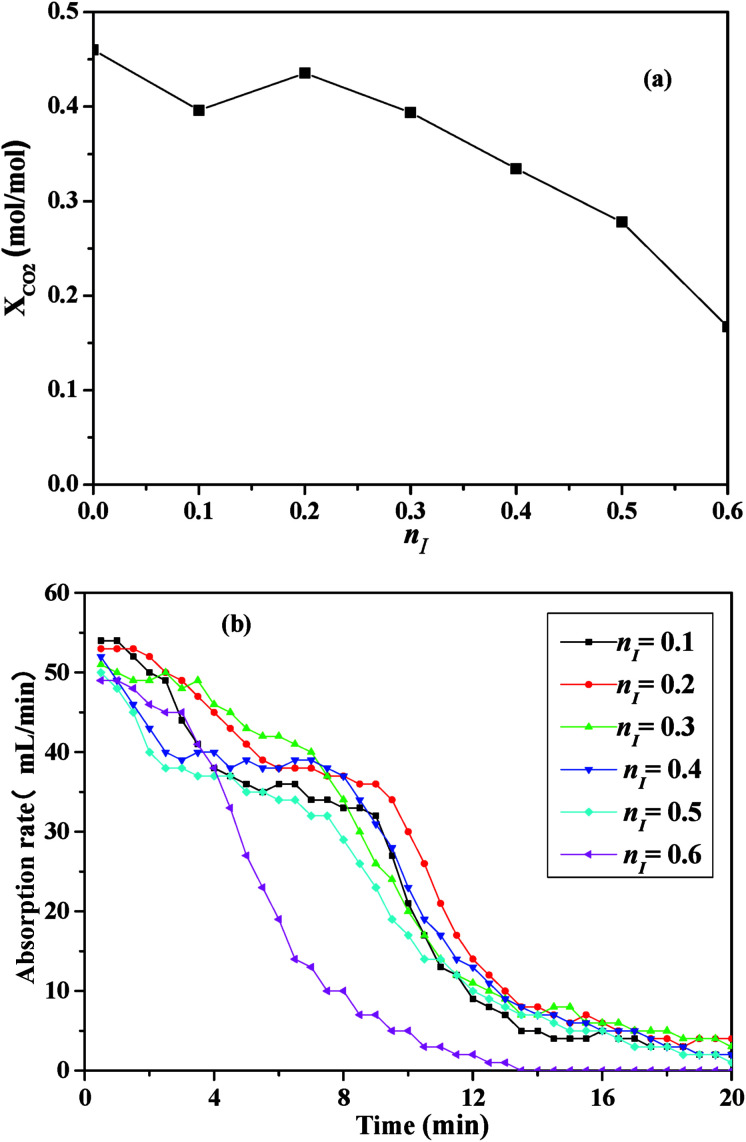
CO_2_ absorption performance of the absorbents with different *n*_I_ at 303 K: (a) the absorption capacity; (b) the absorption rate.

Fig. 3 illustrated the effects of *n*_I_ on CO_2_ absorption performance at 303 K under 1 bar.

As shown in Fig. 3(a), with the increase of *n*_I_, CO_2_ absorption capacity increased initially, and reached a maximum of 0.4554 mol per mol (contrasting with a value of 0.4601 mol CO_2_ per mol MEA measured at the same condition) when *n*_I_ was 0.2, then decreased, which suggested there was an optimum *n*_I_ in the absorbent for CO_2_ absorption. In Fig. 3(b), as time went on, the absorption rate of all absorbents decreased rapidly within 12 minutes, and then decreased gently. With the increasing of *n*_I_, CO_2_ absorption rate increased firstly and then decreased, which suggested that an optimal *n*_I_ also existed.

CO_2_ absorption performance was influenced by the viscosity of the absorbents, the imidazole ring content and the amine group content in the absorbents.^35,36^ CO_2_ absorption capacity was defined as the amount of CO_2_ absorption per mol amine in this work, thus the variation of CO_2_ absorption performance was mainly caused by the viscosity and the imidazole ring content of the absorbents. On the one hand, [NH_2_e-mim][BF_4_] concentration increased as *n*_I_ increased and consequently increased the viscosity of the absorbents just as Fig. 2(b) shown, which militated against the contact of CO_2_ and the absorbents. On the other hand, the increase of *n*_I_ should cause the increase of the imidazole ring content in the absorbent, which benefits CO_2_ absorption.^36^ The comprehensive effects of the factors caused that CO_2_ absorption performance increased and then decreased with the increase of *n*_I_.

**Fig. 4:**
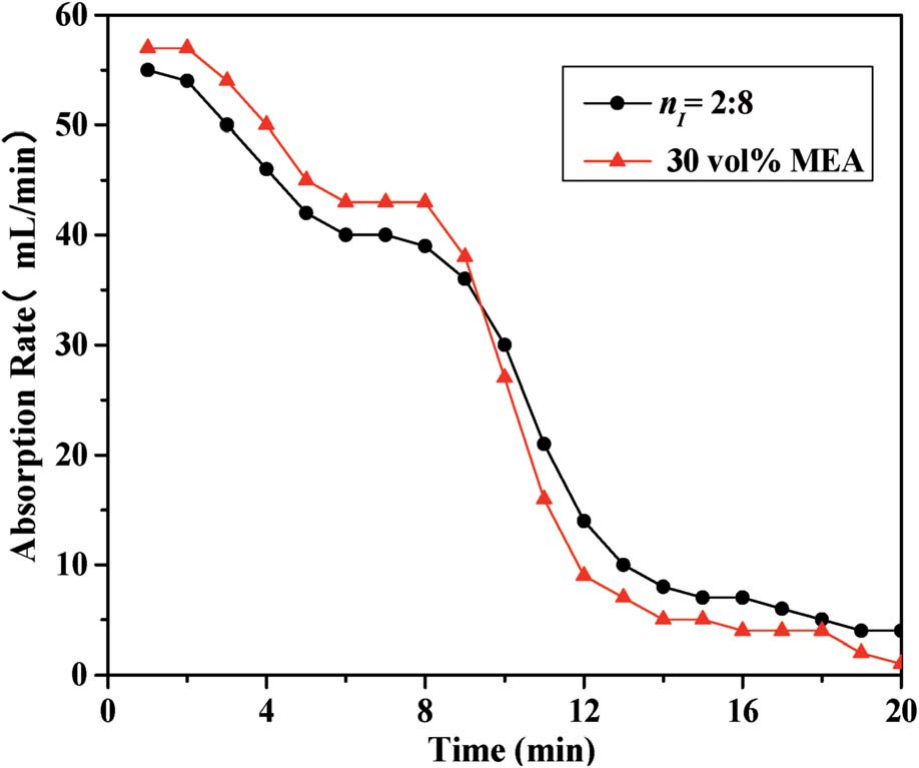
Comparison of CO_2_ absorption rate between the absorbent with *n*_I_ of 0.2 and the 30 vol% MEA solution at 303 K.

As illustrated in Fig. 3, the absorbent prepared with *n*_I_ of 0.2 (I/M_2:8_) showed the best absorption performance. Thus, a comparison between the absorption rate of the I/M_2:8_ and the MEA solution at 303 K under 1 bar had been made in Fig. 4. At the early stage (0–10 minutes), the aqueous MEA solution showed higher absorption rate than the mixture of [NH_2_e-mim][BF_4_] and MEA. After ten minutes, the mixture absorbent showed a higher absorption rate, which suggested that an moderate amount of [NH_2_e-mim][BF_4_] would contribute to increasing CO_2_ absorption rate at the later stage mainly.

Considering that the mixed absorbent with the I/M_2:8_ showed the best absorption performance, the mixed absorbent mentioned below was prepared with this ratio.

### Effects of the temperature on the absorption performance

**Fig. 5:**
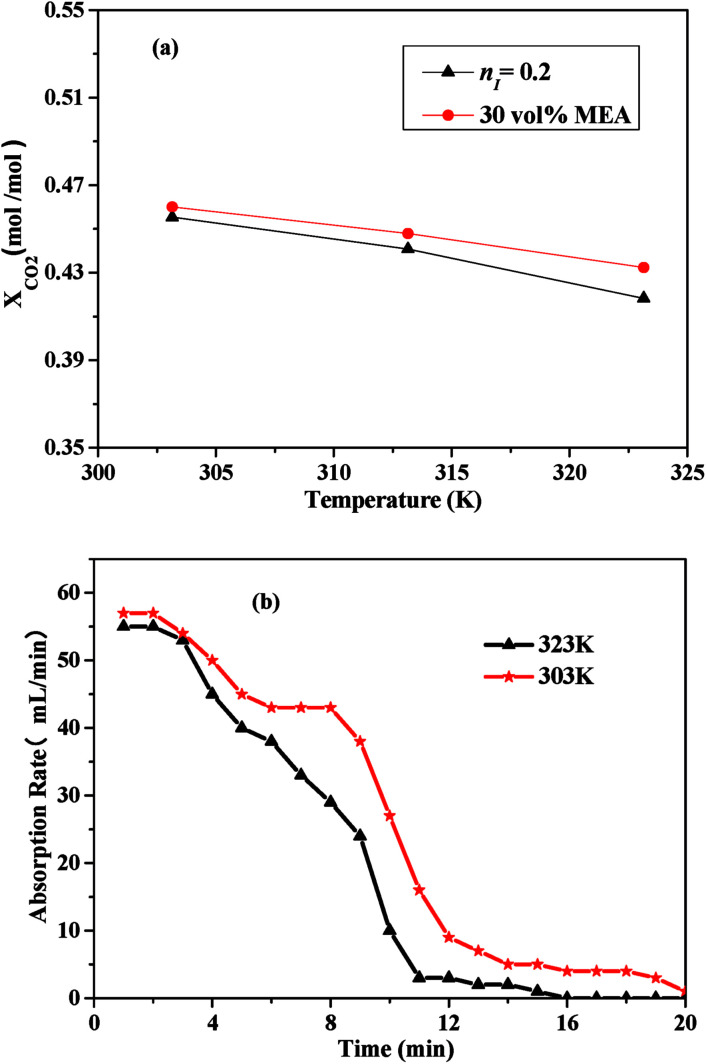
CO_2_ absorption performance of the I/M_2:8_ absorbent: (a) CO_2_ absorption capacity *vs.* temperature; (b) CO_2_ absorption rate *vs.* time at 303 K and 323 K.

Fig. 5 showed the effects of the temperature on CO_2_ absorption capacity and rate of the 30 vol% MEA solution and the I/M_2:8_. According to Fig. 5(a), the CO_2_ absorption capacity decreased as the temperature increased. The comparison between the CO_2_ absorption capacity of the mixed absorbent and the MEA solution illustrated that CO_2_ absorption capacity of the latter was comparable to the former. From Fig. 5(b), the CO_2_ absorption rate decreased with the temperature ranged from 303 K to 323 K. Absorption equilibrium could be reached a little bit earlier at a higher temperature of 323 K, which might due to a lower CO_2_ absorption capacity.

Therefore, both CO_2_ absorption capacity and CO_2_ absorption rate of the absorbents decreased with increasing of temperature. Similar result could be found in the previous studies.^37–39^

### Effects of the temperature on the desorption performance

**Fig. 6:**
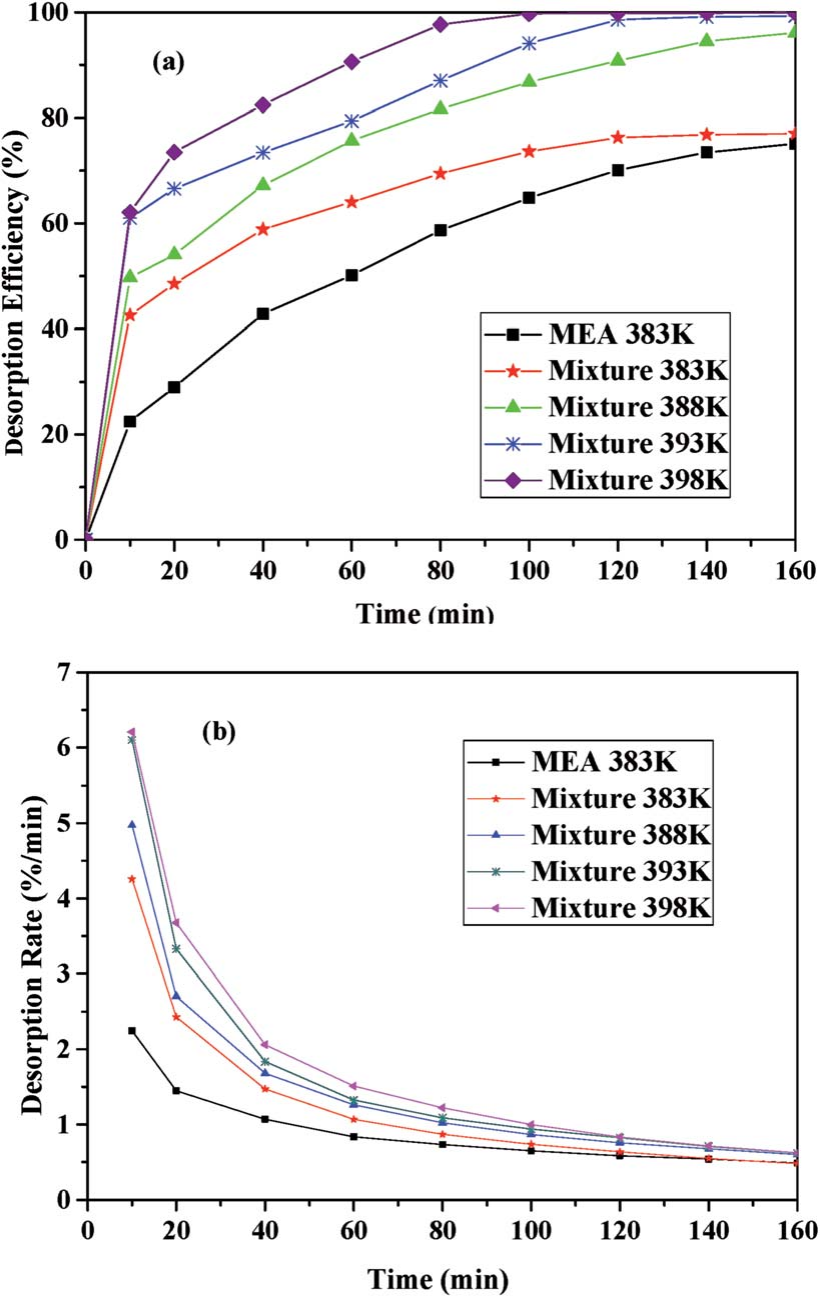
CO_2_ desorption performance the I/M_2:8_ absorbent at different temperature: (a) the desorption efficiency *vs.* time; (b) the desorption rate *vs.* time.

Fig. 6 presented the desorption performance of the I/M_2:8_ at different temperature and the desorption performance of 30 vol% MEA at 383 K under 1 bar. As shown in Fig. 6(a), all the desorption efficiency increased significantly at the beginning (0–40 minutes) and then increased gently until desorption equilibrated. With the increasing of temperature, the desorption efficiency increased. The desorption efficiency of the I/M_2:8_ was much higher than that of the aqueous MEA at the same temperature and it exceeded 50% in 30 minutes. By contrast, the MEA solution needed an hour to reach the desorption efficiency of 50%. The desorption rate decreased intensively first and then decreased slightly until desorption balanced, as shown in Fig. 6(b). With increasing temperature, the desorption rate increased, which followed the same trend as the desorption efficiency. As it could be seen in Fig. 6, the desorption efficiency and the desorption rate at 393 K were close to the values at 398 K. Considering the energy-consumption, 393 K was chosen as the optimum desorption temperature of the mixture with CO_2_ desorption efficiency of 99.31%, which was lower than that of the 30 vol% MEA solution (398 K).

According to Fig. 6, CO_2_ desorption efficiency and rate of the I/M_2:8_ were higher than that of the aqueous MEA at the same conditions, which suggested that the mixture absorbents had a lower energy consumption for regenerations compared with the aqueous alcohol. The energy-consumption of CO_2_ desorption involved three parts: the energy for the increase of the mixture temperature, which was the product of the mole quantity, molar heat capacity and the differential of the temperature; the energy for the escape of CO_2_, which was equal to the absorption heat of CO_2_; and the energy for the vaporization of the absorbent.^1^ The regeneration temperature of the functional ILs was from 263 K to 343 K,^40^ which was lower than that of the alcohol amine. Thus, the ILs needed less energy for the increase of the temperature. The absorption heat depended on the heat of the reaction between the absorbents and CO_2_, which were nearly the same for these two absorbents. Therefore, the energy for the escape of CO_2_ from the two absorbents showed little change. The regeneration temperature of the MEA solution was higher than ILs, accordingly, the vaporization of water and MEA needed more energy. Consequently, CO_2_ desorption of ILs system required less energy than the MEA system, which suggested that the ILs was an economical solution for CO_2_ capture.

### Stability performance of the absorbent

**Fig. 7:**
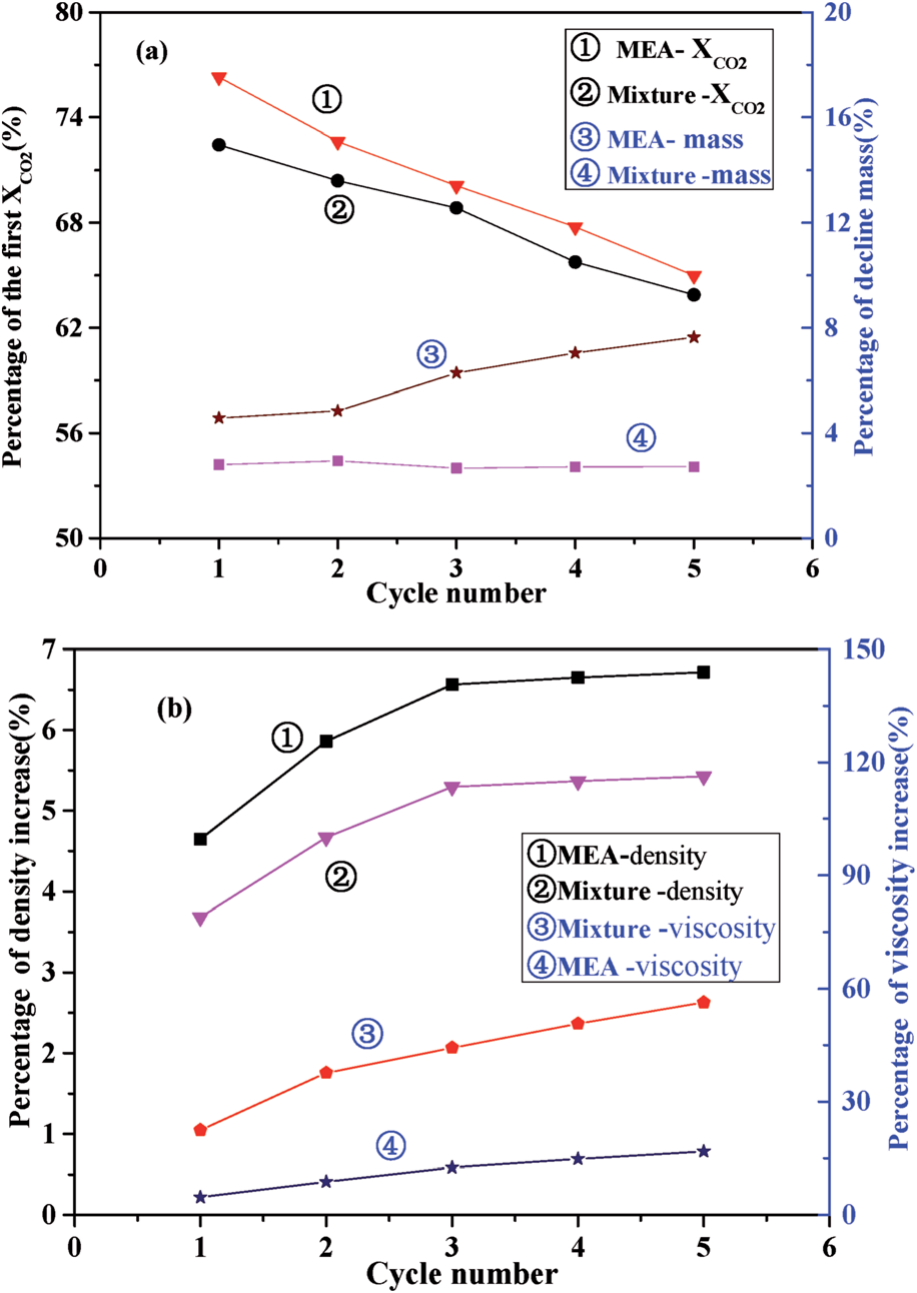
Properties of the I/M_2:8_ absorbent during 5 times cycle: (a) CO_2_ adsorption capacity as a percentage of the first time and percentage of quality decline compared with the first time; (b) percentage of density and viscosity increase compared with the first time.

To examine the stability performance of the absorbents, properties including CO_2_ adsorption capacity, mass loss, density and viscosity of the MEA solution and the I/M_2:8_ were circularly tested for 5 times. The absorption temperature was 303 K, and the time of duration was 30 min. The desorption temperature of MEA solution was 398 K, while the desorption temperature of I/M_2:8_ was 393 K, and the time of duration was 120 min. Fig. 7 showed the properties of the absorbents in each cycle.

As shown in Fig. 7(a), the values of CO_2_ absorption capacity of the MEA solution and the I/M_2:8_ decreased gradually in five absorption–desorption cycles. And the decrease percentage of CO_2_ absorption capacity of the I/M_2:8_ were slightly less than those of MEA. However, the mass weight loss of the two absorbents in the 5 cycles showed different trends. The weight loss percentage of the I/M_2:8_ was 3% relative to the first circle after one cycle, and then the weight was a small loss during the next four cycles. By contrast, the mass of MEA system decreased more obviously, and the percent of the decline values increased as the number of the circles went up. The difference of the two absorbents might be caused by the water and MEA which was more volatile at a higher temperature of 393 K. To verify the reason of the phenomenon, the density and the viscosity of the absorbents were measured respectively in each circle. The results were shown in Fig. 7(b).

As illustrated in Fig. 7(b), as the number of absorption–desorption cycles went up, the density of the absorbents increased gently and then became invariant after the third cycle. In the second cycle, the density of the I/M_2:8_ increased about 3.5% relative to the first circle, while the increase amount of the MEA solution was about 4.5%. The viscosity of the absorbents also increased as the number of absorption–desorption cycles went up. The viscosity of the MEA solution increased in a range of 2–5% with the increase of the cyclic times. And for the I/M_2:8_, it increased about 12% relative to the first circle, and the growth rate slowed down after the second circle with the increase amount of about 5%. The viscosity value of the mixture was in a range of 4–10 mPa s at 303 K, which was still a moderate viscosity for CO_2_ adsorption. From the above results, it suggested that the mixture of ILs and MEA showed a better stability of density and a larger change of viscosity, which mitigated the loss of volatile MEA and water in the absorbents.

According to Fig. 7, it was concluded that the mixture of IL and MEA had a better stability in CO_2_ adsorption capacity, mass loss and density and had a better thermodynamic stability than the MEA solution.

## 4. Conclusions

In this work, absorbents with excellent CO_2_ capture performance were developed by mixing 30 vol% MEA solution with an ionic liquid [NH_2_e-mim][BF_4_]. The density and the viscosity of the absorbents increased with the increase of *n*_I_. The addition of MEA solution reduced the viscosity of absorbents to no more than 10 mPa s.

The absorbent exhibited a rather excellent CO_2_ capture performance when *n*_I_ was 0.2: (1) a moderate [NH_2_e-mim][BF_4_] could contribute to increasing CO_2_ absorption rate at the later stage; (2) CO_2_ absorption capacity of the I/M_2:8_ could comparable with the 30 vol% MEA solution. It contributed to advance the CO_2_ absorption process to increase temperature, however, CO_2_ absorption capacity and rate decreased.

Both CO_2_ desorption efficiency and CO_2_ desorption rate increased with increasing temperature. And CO_2_ desorption efficiency and rate was higher than the 30 vol% solution at the same conditions. Considering the energy-consumption, 393 K was chosen as the optimum desorption temperature, which was 5 K lower than the MEA solution.

Compared with MEA solution, the absorbent of I/M_2:8_ showed a better cyclic stability. The reason was that the addition of [NH_2_e-mim][BF_4_] into the MEA could improve the thermodynamic stability of the absorbent.

## Conflicts of interest

There are no conflicts to declare.

## Supplementary Material
